# Exploring stakeholders perspectives on TB contact investigation in Cali, Colombia: a qualitative study

**DOI:** 10.3389/fpubh.2023.1204862

**Published:** 2023-07-25

**Authors:** Jairo E. Palomares Velosa, Jorge E. Figueroa Gómez, Claudia N. Rojas Zúñiga, Gustavo Díaz, Beatriz E. Ferro, J. Lucian Davis, Lauretta E. Grau

**Affiliations:** ^1^Centro Internacional de Entrenamiento e Investigaciones Médicas – CIDEIM, Cali, Valle del Cauca, Colombia; ^2^Universidad Icesi, Cali, Valle del Cauca, Colombia; ^3^Secretaría de Salud del Distrito, Cali, Valle del Cauca, Colombia; ^4^Departamento de Ciencias Básicas Médicas, Universidad Icesi, Cali, Valle del Cauca, Colombia; ^5^Department of Epidemiology of Microbial Diseases, Yale School of Public Health, New Haven, CT, United States; ^6^Pulmonary Critical Care and Sleep Medicine Section, Yale School of Medicine, New Haven, CT, United States

**Keywords:** contact investigation, tuberculosis, qualitative research, barriers and facilitators, communications, TB test uptake

## Abstract

**Introduction:**

Contact investigation is a proven intervention for tuberculosis (TB) case finding and prevention. Although widely endorsed by national public health authorities and the World Health Organization, many countries struggle to implement it effectively. The objective of the study is to describe and characterize the barriers and facilitators of TB contact investigation in Cali, Colombia from the perspective and experience of the key stakeholders involved.

**Methods:**

We collected data from group discussions during two workshop sessions with clinic and public health staff involved in TB contact investigation (June 2019 and March 2020 respectively) and semi-structured interviews with TB cases and their household contacts (July 2019 to April 2020). We undertook an inductive thematic analysis with the RADaR technique to characterize the barriers and facilitators of the TB contact investigation process.

**Results:**

The two workshops included 21 clinics and 12 public health staff. We also conducted 26 semi-structured interviews with TB cases and their household contacts. Using thematic analysis, we identified four common themes: Healthcare Operations, Essential Knowledge, Time Limitations and Competing Responsibilities, and Interpersonal Interactions. The main barriers to conducting household visits were low data quality, stigma and mistrust, safety concerns for health workers, and limited resources. The main barriers to TB uptake by contacts were competing responsibilities, low TB risk perceptions among contacts, and difficulty accessing diagnostic tests for contacts. In contrast, good communication and social skills among health workers and accurate TB knowledge facilitated successful household visits and TB test uptake, according to key stakeholders.

**Conclusion:**

This study provides a deeper understanding of TB contact investigation barriers and facilitators in a high-prevalence urban setting in a middle-income country from the perspective and experience of key stakeholders. The study shed light on the barriers that hinder household contacts engagement and TB test uptake such as issues of systemic capacity and TB knowledge. Also, highlighted facilitators such as the importance of interpersonal communication skills among health workers in the public and private sector. The insights from this study can serve as a valuable resource for public health organizations seeking to enhance their contact investigation efforts and improve TB control in similar settings.

## 1. Introduction

Tuberculosis (TB) is a subacute or chronic infectious disease caused by *Mycobacterium tuberculosis* that affects approximately 10 million people annually (1). Despite efforts to reduce its incidence, TB remains a major global cause of morbidity and mortality.

Early identification of new cases and prompt access to treatment are essential to reducing TB incidence. According to the World Health Organization (WHO), the global TB treatment coverage rate in 2021 was only 61% (1). TB contact investigation can help improve this figure by identifying undiagnosed individuals with TB and linking them to treatment while also providing those exposed to TB but do not yet have the disease with effective preventive strategies (1, 2). In 2012, the WHO formally recommended active household contact investigation as the routine protocol for high-burden countries (3). Notwithstanding, contact investigation programs face barriers that decrease their potential effectiveness. In large cities in Latin America, these barriers include geographical and social diversity, poverty, urbanization and migration, and decentralized and highly privatized healthcare systems (4).

Although generalizations across different settings can be challenging, studies have identified various barriers to TB contact investigation in low and middle-income settings. First, limited resources such as lack of access to healthcare services, inadequate healthcare infrastructure, shortages of trained professionals, and diagnostic tools (5–9). Second, socioeconomic factors such as poverty, unemployment, and inadequate housing conditions contribute to difficulties in locating and reaching household contacts, while limited financial resources and inadequate social support systems complicate individuals’ adherence to TB testing (10–13). Third, behavioral factors including cultural beliefs, stigma, low health literacy leading to low risk perception and misconceptions about TB, and language barriers create additional obstacles to effective communication and understanding during contact investigation (8, 14–17). Recognizing and addressing barriers in context is crucial to improving TB control efforts and developing more effective contact investigation strategies.

Colombia is considered an intermediate-prevalence country for TB. However, only two-thirds of all incident TB cases are reported to the public health authorities (1). In 2022, there was a 10% increase in TB incidence in Colombia compared to 2019; 2020 and 2021 had reduced reporting rates due to COVID-19 prioritization of public health resources (18). National regulations and guidelines require that newly diagnosed TB cases be reported, with information on possible contacts, to a centralized national surveillance system (SIVIGILA) (19). The local public health authority (Secretariat of Public Health; SoPH) aggregates these reports and initiates the official contact investigation process. Current guidelines recommend screening and testing for active TB disease and referral for evaluation and treatment of latent TB infection. Currently, Colombia’s contact investigation policies for TB is based on active surveillance strategies including household visits (19). However, in Cali, the local yield of contact investigation, defined as the proportion of new TB cases notified to public health authorities among all contacts screened, is less than 1% (20) and in marked contrast to the expected rate of 3.1% for lower-middle income settings (7, 21).

As part of our continuous efforts to enhance the implementation of TB contact investigation, we conducted a qualitative study in collaboration with key stakeholders. This study aimed to identify and characterize the barriers and facilitators of TB contact investigation in Cali, Colombia. By engaging with stakeholders including clinic staff, SoPH personnel, and TB cases along with their household contacts, we sought to gain valuable insights into the factors influencing the engagement of household contacts and their uptake of TB testing.

## 2. Methods

### 2.1. Study setting

The study was conducted in Cali, a city of 2.5 million inhabitants and Colombia’s third-largest metropolitan area. In 2020, the estimated local TB incidence was 40 per 100,000 (22), almost double the national incidence (23).

The research team has established a longstanding collaboration with the SoPH in various previous projects related to TB. Over the years, we have worked closely with the SoPH to investigate and address key challenges in TB control, ranging from contact investigation strategies to improving engagement and testing among household contacts.

### 2.2. Study sample

The selection of participants for this study was based on their roles, experiences, and perspectives relevant to the research objectives. The study sample comprised three groups with different roles on the CI process. These included:

1. Clinic staff: this group consisted of healthcare workers at hospitals, clinics, or health posts, who are involved in the delivery of TB healthcare services including diagnosis, treatment, and data collection monitoring, or reporting.
2. SoPH staff: this group included public health officials and community health workers (CHWs) who are part of the TB prevention and contact investigation program.
3. Cases and contacts: this group comprises individuals who are TB cases and their household contacts. They are the primary focus of the contact investigation process.

The data collection methods consisted of workshops (including group discussions), and in-depth interviews. Workshop participants were clinic staff involved in TB healthcare services and SoPH staff from the TB contact investigation program. Two separate workshops were conducted with these two groups to gather their perspectives and experiences through group discussions. Both cases and their household contacts were interviewed to gain insights into their interactions with clinic and SoPH staff and understand their decisions regarding seeking TB testing.

### 2.3. Recruitment and data collection procedures for the clinic and SoPH staff workshops

The SoPH TB program head initiated the recruitment of clinics’ staff by issuing official invitation letters to directors of the largest public and private healthcare institutions that offer TB services. This letter briefly explained the purpose of the study and extended an invitation to clinics staff involved in TB-related services to attend a one-day workshop on how to improve CI in Cali. Additionally, the TB program staff of the clinics were contacted through follow-up phone calls to confirm their participation and schedule. The first workshop, held at a local university (Icesi) in June 2019, utilized this approach.

For the second workshop, the research team (GD, BF, and CR) sent verbal and email invitations to all SoPH staff actively involved in TB contact investigation activities. This second workshop took place at a local university (Icesi) in March 2020, mirroring the format of the first workshop.

Two researchers, a sociologist (JF) and a psychologist (CR) experienced in group facilitation, led the workshops. Each lasted about 6 hours and included multiple group discussions and breakout sections (24). The objectives of the workshops were threefold: firstly, the participants were tasked with identifying all the necessary steps for the successful completion of contact investigation. Secondly, they were encouraged to describe the barriers and facilitators associated with each of these steps from their own perspectives. Lastly, the participants were encouraged to propose strategies aimed at overcoming these barriers and enhancing the facilitators. Audio recordings were made for the group discussions during the main sessions of the workshops, excluding the breakout sessions. However, it is important to note that the recording of the first workshop was unsuccessful, resulting in the absence of audio data for that session. Instead, facilitators’ notes were diligently taken during and immediately after the workshop to capture the relevant information. The audio recording for the second workshop was successfully obtained and subsequently transcribed verbatim, ensuring a comprehensive record of the discussions.

### 2.4. Recruitment and data collection procedures for the cases and contacts group

To recruit the participants from the cases and contacts group, we generated a random sample from a database of contact information for all cases diagnosed with TB in 2017 and their household contacts (provided by the SoPH). To ensure heterogeneity, the sample was stratified by insurance type (public or private), residential zone within Cali, and records of contacts completing referral for TB testing. The potential participants were initially contacted by phone. After acceptance, an appointment for the study interview was scheduled. We excluded participants who were incarcerated or homeless.

Between July 2019 to April 2020, we conducted a total of 26 semi-structured interviews with participants in the cases and contacts group. Initially, we conducted the interviews at participants’ preferred location (mostly at their homes). However, due to the COVID-19 pandemic that emerged in March 2020, we conducted the remaining (7) interviews via telephone in compliance with public health policy/recommendations. The content of the interview guide was directed to explore participant experiences while receiving TB-related services including: (i) perceptions of interactions with clinic staff, and SoPH, (ii) perceptions and experiences related to the household visit, and (iii) their reasons and motivation for accepting or declining the household visits, and reasons why the contact did or did not complete TB testing. All interviews were conducted by researchers trained in qualitative interviewing methods (JF, CR, and JJ). The interviews were audio-recorded, subsequently transcribed verbatim, and continued until data saturation was achieved.

### 2.5. Data analysis

The data analysis did not adhere to a specific theoretical framework or approach but incorporates methodological elements that bear similarities to phenomenology and grounded theory. The analytic approach was inductive and iterative, consisting of two stages. The data analysis team for the first stage included a sociologist/anthropologist (JF) a psychologist (CR), and two microbiologists (GD and BF), all with advanced degrees in their respective fields. This stage involved a qualitative iterative data exploration with inductive open and axial coding that culminated in a 30-page report to the Colombian Ministry of Science, Technology, and Innovation.

The second stage of data analysis involved an expanded team consisting of two additional researchers with doctoral degrees (JP epidemiology; LG behavioral psychology) and experience in qualitative data analysis. The second stage goal was to further synthesize the report using thematic analysis (25, 26) and the Rigorous and Accelerated Data Reduction (RADaR) technique (27). Briefly, the RADaR technique involves the develop all-inclusive data tables in spreadsheets and iterative reductions in the data. The data analytic team for the second stage met weekly and data-reduction decisions were based on unanimous agreement.

## 3. Results

### 3.1. Description of the study sample

The first workshop was attended by 21 participants from 14 clinics (out of 18 invited). Seven of the clinics were private and seven were public. The profiles of the participants included project managers, supervisors, program coordinators, area coordinators, professional nurses, and one auxiliary nurse. Similarly, 12 members of the TB section of Cali’s SoPH, agreed to participate and attended the second workshop, including 10 CHWs, one physician, and one professional nurse.

The 26 interviews included 15 individual interviews (2 individual cases and 13 individual contacts), and 11 group interviews [7 cases and their contact(s), and 4 contacts only], for a total of 40 participants (9 cases and 31 contacts). The median age of this group was 50 (range 24–80). About two-thirds were female, and most were of White-Mestizo race. Most had private insurance (67.5%). Most participants (83%) resided in lower-income neighborhoods (Table 1).

**Table 1:** Description of the cases and contacts participant group sample (*n* = 40).

Characteristics	Total
Gender	
Female	25 (63%)
Male	15 (37%)
Age (years)^a^	53 (24–80)
Race	
Indigenous	1 (2.5%)
African Colombian	1 (2.5%)
White-Mestizo	38 (95%)
Type of health insurance	
Contributory (private)	27 (67.5%)
Subsidized (public)	11 (27.5%)
Other^b^	2 (5%)

a Mean (range).

b Special, prepaid, or traditional medicine.

### 3.2. Context-specific steps of TB contact investigation

**Figure 1:**
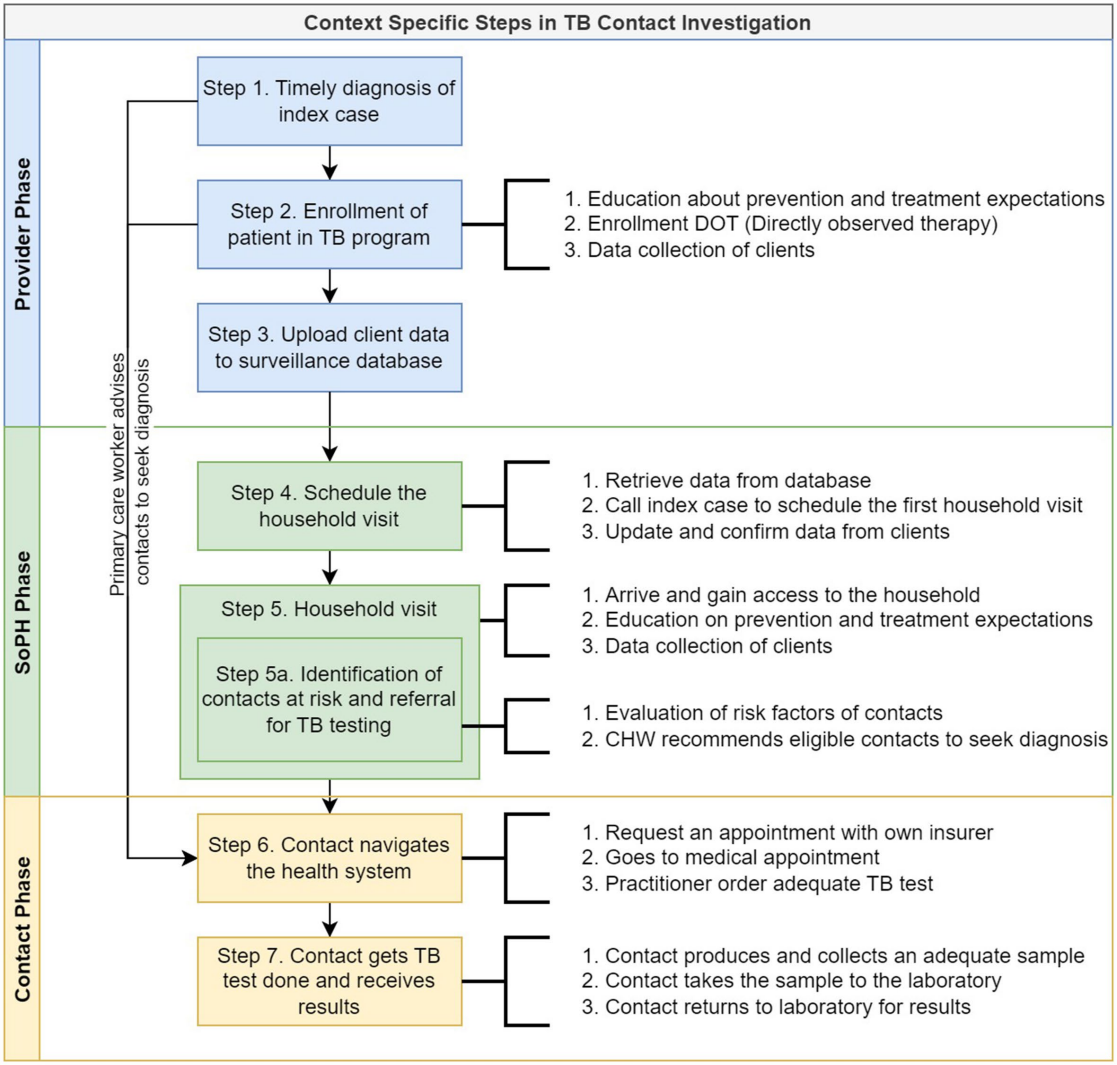
Context-specific steps and related activities of the TB contact investigation process as described by the stakeholders involved. SoPH, Secretary of Public Health; TB, tuberculosis; cases and contacts cases and contacts.

As a secondary product of the group discussions during the workshop, the participants summarized the TB contact investigation steps in Cali, which we divided into the provider, public health, and contact phases (Figure 1). Identifying the steps was necessary in part to assess the fidelity of the implementation of national regulations based on WHO guidelines (results not included), but also to facilitate identification and characterization of the barriers and facilitators in every step.

### 3.3. Thematic analysis

The data analysis identified four themes and ten sub-themes (Table 2). The Healthcare Operations theme concerned characteristics at multiple levels of the healthcare system (i.e., national, regional, and local) that can influence the outcomes of contact investigation. The Essential Knowledge theme concerned information required to complete a given step of contact investigation. The Time Limitations and Competing Responsibilities theme included any situation or activity in which a required activity could not be completed given the available time and the need to fulfill other duties. The Interpersonal Interactions theme focused on how one’s relationships with others could impact contact investigation outcomes.

**Table 2:** Barriers and facilitators of TB contact investigation identified by study participants.

Themes & Sub-themes	Barriers	Facilitators
Healthcare operations		
Data quality and inter-institutional coordination	Provider: missing or inaccurate data reported to the surveillance system impedes tracking of contacts SoPH: missing data impedes the scheduling of household visits SoPH: suboptimal local inter-institutional cooperation limits progress in solving data quality issues Contact: inadequate or missing communication between insurers impedes the uptake of TB testing.	None identified
Resource needs	SoPH: lack of specific guidelines and scripts for scheduling and household visit procedures affect scheduling success. SoPH: lack of dedicated phone lines for contact investigation-related tasks creates inconveniences for CHWs	None identified
Access to households	SoPH: missing data hinders scheduling efforts and increases the risk of visit rejection or absence of some household members SoPH: safety concerns impede access to households in some neighborhoods	SoPH: collaboration with community leaders promotes access to households in unsafe neighborhoods
Essential knowledge		
General TB knowledge	Provider: lack of TB awareness by physicians causing delayed diagnosis Provider: misinformation among staff causing negative interactions with index cases Contact: low-risk perception decreased the likelihood of contacts seeking TB diagnosis Contact: misinformation about transmission within the household resulted in unnecessarily harsh preventive measures against TB cases.	Provider: index cases disseminate accurate information to their contacts, increasing test uptake Contact: seeking diagnostic behaviors was reported among those with better TB knowledge and good TB knowledge transfer interactions
Skills needs for Clinic and SoPH staff	None identified	SoPH: knowledge of the structure of the healthcare system to find or correct patient information SoPH: good communication skills increase the likelihood of acceptance of household visits and associated tasks
Time limitations and competing responsibilities		
Time constraints of Clinic and SoPH staff	Provider: time limitations for physicians affect data quality SoPH: multiple job responsibilities complicate scheduling household visits	None identified
Competing demands for Contact	SoPH: C/co competing demands prevent the scheduling of household visits Contact: competing demands related to work or caring for relatives impedes TB uptake| Contact: time and effort to obtain a prescription for TB testing for contacts	None identified
Interpersonal interactions		
Stigma	Provider: stigmatizing experiences with clinic staff could discourage further interaction with the clinic or SoPH staff SoPH: anticipated stigma can prevent acceptance of household visits	None identified
Trust	None identified	SoPH: continuity of CHW for scheduling and visits increased the likelihood of acceptance of household visits Contact: follow-up visits, particularly by a trusted CHW, encouraged contacts to seek TB testing

The table organizes barriers to and facilitators of the TB contact investigation process for each theme and sub-theme identified. CHW, community health workers; SoPH, Secretary of Public Health; TB, tuberculosis.

### 3.4. Theme 1: healthcare operations

We identified three sub-themes: Data Quality and Inter-Institutional Coordination, Resource Needs, and Access to Households, each of which is described further below.

#### 3.4.1. Data quality and inter-institutional coordination

This sub-theme focused on national or local policies or institutional procedures that could promote or impede smooth and timely progression through the contact investigation process.

Missing or inaccurate data from TB cases was a common barrier during the Provider and SoPH Phases. CHWs attributed poor data quality to inefficient data systems and the low priority given to data management among many other clinic tasks. For example, TB-related surveillance data is centrally managed through the national surveillance system platform, and SoPH staff lack the authority to correct data errors. As a result, SoPH officials were forced to try to contact clinic staff to clarify or update the correct information in the database, with unreliable results:

"When we find out that there is missing or inaccurate information about a case, we then call the providers (to solve missing information). Sometimes due to their own tasks, or … sometimes they just evade you and do not want to give you the information." (CHW 04)

Another possible source of access to diagnostic delays is Colombia’s individual-level insurance system in which access to testing often varies widely between individuals and even within households. CHWs and cases and contacts noted that the steps required for the contacts to obtain a test order from their provider complicate test-seeking behaviors and delay access to testing. In one scenario, a contact reported that a physician refused to provide a test order, saying that each person had to contact their insurer for them to obtain a test order. Conversely, having the same insurer for all family members facilitated the process.

"Yes, of course, when the whole family group has the same (insurer), it (getting tested) is easier." (CHW 10)

#### 3.4.2. Resource needs sub-theme.

This sub-theme focused on how additional or reallocated resources could improve contact investigation. For example, the lack of a standardized script for CHWs to use when scheduling visits posed a potential barrier. One CHW indicated that scripts did not exist, while another quipped, *“Yes, we all have our own script.”*

Lacking a dedicated SoPH telephone line for scheduling household visits was another barrier, in that it forced SoPH staff to use their own mobile phones for work activities. CHWs occasionally received return calls outside work hours including requests for information or health services outside the scope of contact investigation that infringed on personal time or threatened their personal safety.

"This is a problem (using our phones), sometimes they (cases and contacts) call at 6 or 7 pm to ask for (information about medical services) … (Another CHW added)" … It happened to me that (a contact) harassed me by phone and (stalked) me for a while." (CHW 08 and CHW 09)

#### 3.4.3. Access to households sub-theme

This sub-theme concerned the challenges faced in reaching households to conduct home visits. As such, it primarily focused on potential barriers, although we identified some potential facilitators.

Difficulties obtaining the correct phone numbers for cases and contacts sometimes necessitated arriving at homes without formally scheduling a visit in advance. Unannounced visits sometimes upset the person (s) receiving the visit and could negatively impact interactions and the ability to complete the evaluation of all household contacts for TB testing.

"Sometimes, you just get there (without previous notification), sometimes they accept the visit, other times they reject it if they see that we are from the SoPH." (CHW 04)

CHWs mentioned other household access barriers, including unsafe neighborhoods, poorly maintained roads, rugged terrain, and lack of transportation to some city areas. To address safety concerns, CHWs sometimes arranged to interview TB cases at nearby clinics or other public locations, but this types of arrangement prevented concurrent evaluation of household contacts. Another strategy to address safety concerns was to work with local community leaders.

"(To access the households, we) need to walk (long distances), hire transportation … depending on the location, there may be safety concerns. Then we need to be accompanied by police or local leaders." (CHW 04)

### 3.5. Theme 2: essential knowledge

In this theme, we identified two sub-themes, General TB Knowledge and Skills Needs for Clinic and SoPH Staff. Evidence of this theme occurred in all three phases. Each sub-theme is defined below.

#### 3.5.1. General TB knowledge sub-theme

All participants considered knowledge about TB pathophysiology, diagnostic procedures, transmission risk, prevention measures, and treatment options essential to all phases of the contact investigation cascade.

Sometimes, healthcare providers may not include TB in the differential diagnosis, which can delay testing. One contact felt that the person with TB had not been diagnosed promptly because the providers were not knowledgeable about TB screening and diagnosis:

"None of them (clinic providers) were certain that it was TB because he (the person with TB) had no symptoms." (Contact 11)

Another example where insufficient awareness of TB risks influenced the contact investigation process was observed when cases and contacts refrain for seeking testing due to the absence of symptoms:

"I was not (even) worried (about having active TB) because I did not have any symptoms." (Contact 31)

However, household contacts also described instances where having accurate TB knowledge facilitated the contact investigation process. TB education and counseling that occurred at enrollment in the TB program allowed TB cases to share this knowledge with others subsequently. Relatives also sometimes accompanied TB cases during enrolment, providing another opportunity to disseminate useful TB information to others in the household.

"My dad explained to me about tuberculosis, the recommendations, and the (assessments), based on what he was told at the doctor’s (office)." (Contact 15)

In addition to the importance of having accurate information about TB, participants described instances where misinformation about TB transmission risk and prevention could promote fear and stigma in households of TB cases. Cases, contacts, and CHWs provided examples such as incorrect routes of transmission or prevention practices that led to stigmatizing experiences or engaging in ineffective prevention strategies.

"When we arrived (at the health center), and we provided the history (clinical records document), the girls (staff) were all scared. The chief nurse made us leave … (they said) that he (the person with TB) could not be on the street like that, that he had to wear a high-efficiency mask because he could infect someone. I was told that he could not eat with the (same) utensils that we ate, that he had to be isolated (for) at least 20 days, where he could not be in contact with anyone; then he felt bad and, as we had ignorance of how to handle that type of disease, we believed it was true and isolated him a little." (Contact 06)

#### 3.5.2. Skills needs for clinic and SoPH staff sub-theme

This sub-theme focused on the skills demonstration experiences of clinic and SoPH staff to solve CI issues.

To effectively address problems in data quality, SoPH staff must understand the structure of underlying social networks at the provider institutions in order to obtain and secure the needed information.

" … one solution (to missing data) is to visit the provider clinic and talk directly to the TB program chief (usually a nurse) to obtain the required information … By phone, sometimes they (clinic staff) evade you like 'She is busy, she is not here, ' things like that …." (CHW 11)

CHWs also demonstrated interaction skills when they encountered negative responses from individuals they telephoned, by applying their interpersonal skills to establish positive relationships with cases and contacts and motivate contacts to seek testing. In contrast, experiences where clinic staff demonstrated lack of knowledge and communication skills can negatively influence seeking testing behaviors by contacts.

"Sometimes, (when calling to schedule the visit) they hang up, they refuse the call or even swear at you, this makes establishing the first contact a difficult task." (CHW 05)

Some CHWs also expressed pride in their excellent social and communication skills, noting the need to be sensitive when index cases and their families were distressed by the recent TB diagnosis or other related problems. They considered these communication skills necessary for building trust and improving the chances of scheduling and conducting the household visit. One CHW noted that a common experience when scheduling the initial home visit was that “many times they (TB cases) are frightened, then I try to calm things down …. “.

### 3.6. Theme 3: time limitations and competing responsibilities

Within this theme, we identified two sub-themes, Competing Demands for Clinic Staff and SoPH staff, and Competing Demands for cases and contacts, as defined below.

#### 3.6.1. Time constraints of clinic staff and SoPH personnel sub-theme

This sub-theme included barriers of time constraints for both staff in clinics and public health sector. Work overload for clinic health care workers can place time constraints and may have led some to use shortcuts when completing or updating the necessary forms, often undermining data quality.

"The doctors (physicians) are given only 20 minutes (per patient); they have to fill up the (forms), there are a lot of activities, and (there is) too little time." (CHW 01)

CHWs also reported time constraints negatively influencing engagement activities with cases and contacts.

"(Scheduling the visit) is challenging … because we have other activities (job responsibilities), many times the (person with TB) can (meet), but we can’t." (CHW 07)

#### 3.6.2. Competing demands for cases and contacts sub-theme

This sub-theme focused on the competing demands affecting scheduling household visits or seeking and obtaining TB testing. It primarily involved steps in the SoPH Phase and the Contact Phase.

CHWs observed that reaching all contacts in a single household visit was sometimes challenging because of their competing job responsibilities or family obligations. The time required for navigating the healthcare system could also pose a barrier to testing.

“… Usually, when you arrive (at the household), there is only the patient, sometimes there is one person that takes care of her (him) if the patient is an old person or a child or a (special needs) person.” (CHW 05)

"Once, I wanted to go do it (get tested). I couldn't go because my boss didn't give me permission." (Contact 29)

“I (guardian of child contact) did not even want to waste my time trying to get the authorization (for the child’s TB test).” (Index case 05)

### 3.7. Theme 4: quality of interpersonal interactions

This theme included two sub-themes, Stigma and Trust, both central to the success or failure of a given interaction. These issues were especially critical to the success of the provider and SoPH phases.

#### 3.7.1. Stigma sub-theme

This sub-theme focused on how anticipated or experienced stigma could pose a barrier to TB program enrolment and household visits. For example, some participants reported experiencing discrimination by clinic staff, which could lead them to avoid seeking care and feel mistrust towards the health system. Furthermore, CHWs noted that some clinic staff attempted to coerce TB cases into treatment by suggesting that there could be punitive actions from public health officials (i.e., SoPH). This threat could, in turn, undermine effective interactions with household members when CHWs visited their houses.

"They (clinic staff) say (to the patient), like, "If you do not get back to treatment, the SoPH comes, and you will get in trouble." (CHW 12)

Several participants described experiences in which anticipated stigma from family members or neighbors resulted in not disclosing their TB status or refusing a household visit. Some TB cases feared reprisals within their household or community, making it challenging to conduct household visit tasks. Others refused to participate when CHWs identified themselves as representing the SoPH. In those instances, CHWs were obliged to disregard standard SoPH procedures and conduct the visit-related activities elsewhere or at another time.

“She (the person with TB) refused the visit because I had distinctive clothes (SoPH uniform) … she said ‘I will receive the visit if you are wearing regular clothes … my neighbors are very gossipy, and I don’t want them to know (I am sick).’” (CHW 01)

#### 3.7.2. Trust sub-theme

This sub-theme focused on how a lack of trust in the healthcare system posed a barrier to positive interpersonal interactions. CHWs noted that personnel continuity improved trust in the SoPH and facilitated contact investigation. Assigning the same CHW to conduct the follow-up contact investigation activities helped create more opportunities to establish rapport and gain household members’ trust. As such, CHWs were considered as critical to effective contact investigation as the initial household visit.

"When one (CHW) arrives at the house, then you can say ‘Hello, I was the one who called you, remember?’ … so they are more receptive … during the call, you can say the name of the person who is conducting the visit, so they know ahead of time." (CHW 02)

## 4. Discussion

To our knowledge, this is the first qualitative study of TB contact investigation in Colombia. Drawing on the perspective of those involved in TB contact investigation in Cali, Colombia, including clinic staff, public health staff, TB cases, and their household contacts, we have identified a variety of barriers to and facilitators of delivery of this important public health intervention. These findings may inform the development of effective contact investigation strategies not only for Colombia but also for other middle-income countries with low-to-moderate TB incidence and similarly structured healthcare systems (1).

Obtaining a description of the TB contact investigation steps from the key stakeholders was crucial as it allowed study participants to localize possible barriers and facilitators at specific steps. These findings provide firsthand knowledge that can inform a bottom-up approach to improving TB contact investigation in conjunction with local contextual considerations. Hereafter, we discuss the barriers and facilitators of contacts engagement during household visits and TB test uptake by household contacts.

Several barriers were noted to influence the success of engaging and evaluating household contacts. Consistent with the literature (5, 28), inefficiencies within the healthcare system limited the time that clinic staff could spend on tasks other than direct patient care; this, in turn, risked compromising both data accuracy and completeness. These time constraints often required extra efforts by SoPH and clinic staff to resolve data inaccuracies and delayed scheduling of the household visits. Potential interventions to improve the efficiency of data collection and transfer include staff education about the importance of data quality, allotting more time for data entry, and improving data-sharing policies between organizations (29). Policy changes aimed at streamlining the health system procedures for data collection and management could include using electronic systems where possible (29–31).

In our study and as reported elsewhere (14, 15, 32), refusing household visits or recommending unnecessary prevention practices was often based upon inaccurate TB knowledge, anticipated stigma from the local community, or general mistrust of the healthcare system. Stigma has been described as a critical barrier to contact investigation in many settings (14, 15, 32–34), and our findings were also consistent with these findings. A systematic review indicated that strategies such as education and support programs might reduce stigma (35). As described previously (36–38), the data indicated that mistrust in the health system was another barrier to accepting household visits. Future research should seek to understand the nature of this mistrust and whether educational campaigns can reduce this type of mistrust.

Other barriers to conducting home visits that were noted included safety concerns, hard-to-reach locations, and the need for additional resources. The local need for additional resources is a common problem for health programs in low and middle-income countries (7). Interventions that use modern communications technology (29) or mHealth strategies have been shown to improve access to health services in remote or unsafe locations (30, 31). Other strategies that have proven effective in such settings are community-based surveillance programs (39), interventions aided by electronic technologies (29), and increasing capacity (e.g., human resources, infrastructure, and communications) to improve surveillance systems (40).

Concerning the uptake of TB testing by contacts, we identified three main barriers. The first, as described elsewhere (41, 42), was that contacts with low TB risk perception or who were asymptomatic were less motivated to seek testing. This could have been based on inadequate knowledge about transmission risk or lack of early symptom recognition. TB education and public awareness campaigns and targeted outreach to high-risk groups offer strategies to decrease the impact of low risk perception on TB test uptake (43).

Competing responsibilities of cases and contacts was another barrier to seeking TB testing. As described elsewhere (10, 11, 44), cases and contacts were often forced to prioritize their work and family commitments over personal health care needs. Offering flexible testing hours and assistance such as childcare or transportation services may offer one way to reduce the impact of these barriers.

Lack of access to timely diagnosis was a barrier to TB test uptake. Despite the high coverage rate (close to 100%) of health services in Colombia (45), access to essential health services, including diagnostic testing, remains problematic due to the complex nature of the healthcare system and the bureaucracy involved in gaining access to services (46). Consulting physicians’ limited knowledge of and experience with TB was considered a critical factor in delays in diagnosis. TB is a less frequent cause of lower respiratory tract illness than other diseases (22). Consistent with the literature from other settings (6), providers potentially lacked of awareness of local TB epidemiology that then delayed referral for TB testing, diagnosis, treatment, and ultimately the initiation of contact investigation and testing of contacts. Continuing medical education on tuberculosis for providers serving high-incidence communities may improve their ability to diagnose TB cases earlier.

The primary facilitator identified for both outcomes, engagement of contacts through contact investigation and TB test uptake, was the use of good communication and social skills by the clinic and SoPH staff throughout the contact investigation process. For instance, good communication facilitated scheduling household visits and access to hard-to-reach neighborhoods. SoPH staff used good communication and social skills with clinic staff to improve data quality and tracking of cases and contacts and decrease the chances of unannounced household visits. Similarly, positive interactions were thought to increase the likelihood of TB testing uptake by household contacts. These findings are consistent with studies showing that high quality interactions built trust in health workers and improved the uptake of health services by cases and contacts (36, 37, 39, 47–49). Also, good communication between cases and contacts and health workers resulted in better transfer of TB knowledge and increased intentions to undergo TB testing. This is also consistent with the literature in which communication between household members regarding accurate information about transmissible diseases effectively increased health-seeking behaviors and prevented infections (50–52), including TB (53). Our findings suggest that increasing efforts in training and capacity-building programs for SoPH and clinic staff to develop their communication and social skills, including strategies for building rapport with community leaders, engaging with household members in a culturally sensitive manner, and promoting effective communication with healthcare providers (54–56). The study strongly supports previous findings that health workers should aim to establish and maintain trust with patients and communities by demonstrating empathy, respect, and responsiveness to their needs and concerns (57).

Several study limitations should be noted. First, the findings may have limited transferability beyond lower or middle income settings with moderate TB incidence and mixed health systems. Second, although we attempted to mitigate selection bias by randomly selecting potential participants among all TB cases and their household contacts reported in 2017, those who agreed to participate may not have fully represented the target population. Third, social desirability and recall bias may have influenced the trustworthiness of the findings. Finally, due to the poor quality of the first workshop’s audio-recording and the need to rely solely upon the facilitators’ notes, some information may not have been captured and may have affected the interpretation of the thematic analysis.

## 5. Conclusion

The study provides insights into barriers and facilitators of TB contact investigation in Cali, Colombia, from the perspectives of TB healthcare workers at clinics, SoPH staff involved in TB contact investigation, and from TB cases and their household contacts. The main barriers to TB test uptake by contacts were competing responsibilities, low risk perception among contacts, and difficulty accessing diagnostic testing. The main barriers to conducting successful household visits and engaging contacts were data quality, stigma and mistrust, safety concerns for health workers, and limited resources. The primary facilitator was good communication skills; it promoted positive interpersonal interactions, access to and acceptance of household visits, and TB test uptake. Interventions focusing on enhancing TB knowledge and communication skills, improving the collaboration between private insurers and public health authorities in serving cases and contacts, and increasing overall capacity in terms of human resources, physical infrastructure, and technological advancements can greatly enhance the engagement of household contacts and facilitate higher uptake of TB testing. These interventions, in turn, have the potential to significantly improve the detection rates among household contacts and contribute to more effective TB control efforts.

## Data availability statement

The raw data supporting the conclusions of this article will be made available by the authors, without undue reservation.

## Ethics statement

The studies involving human participants were reviewed and approved by Comité Institucional de Ética en Investigación en Humanos of CIDEIM. The patients/participants provided their written informed consent to participate in this study.

## Author contributions

JP was responsible for the analysis and interpretation of results, as well as drafting and writing the manuscript. JF contributed to the conception or design of the research project, as well as the acquisition, analysis, and interpretation of results. CR made significant contributions to the conception and design of the research project, as well as the acquisition of data. GD contributed to the conception or design of the research project and provided critical revision of the manuscript content. BF was involved in the conception or design of the research project and provided critical revision of the manuscript content. JD played a role in the conception and design of the research project, provided critical revision of the manuscript content, and assisted in the interpretation of results. LG was responsible for the analysis and interpretation of results, as well as writing the manuscript and revising the content critically. All authors contributed to the article and approved the submitted version.

## Funding

The data collection and first report were supported by the Ministry of Science, Technology, and Innovation of Colombia-Minciencias (#211780763484CT834-2018) and the Fogarty International Center at the National Institutes of Health (D43 TW006589). The second analysis and further data reduction were also financially supported by the affiliated institutions in the form of time dedicated to this project.

## Conflict of interest

The authors declare that the research was conducted in the absence of any commercial or financial relationships that could be construed as a potential conflict of interest.

## Publisher’s note

All claims expressed in this article are solely those of the authors and do not necessarily represent those of their affiliated organizations, or those of the publisher, the editors and the reviewers. Any product that may be evaluated in this article, or claim that may be made by its manufacturer, is not guaranteed or endorsed by the publisher.
